# Vascular architecture and hypoxic profiles in human head and neck squamous cell carcinomas

**DOI:** 10.1054/bjoc.2000.1325

**Published:** 2000-08-16

**Authors:** K I E M Wijffels, J H A M Kaanders, P F J W Rijken, J Bussink, F J A van den Hoogen, H A M Marres, P C M de Wilde, J A Raleigh, A J van der Kogel

**Affiliations:** Institute of Radiotherapy, Department of Otorhinolaryngology, Departments of Pathology and Maxillofacial Surgery, University of Nijmegen, P.O. Box 9101, Nijmegen, HB, 6500, The Netherlands; Radiation Oncology and Toxicology, UNC School of Medicine, Chapel Hill, USA

**Keywords:** pimonidazole, hypoxia, vascular density, head and neck carcinomas

## Abstract

Tumour oxygenation and vasculature are determinants for radiation treatment outcome and prognosis in patients with squamous cell carcinomas of the head and neck. In this study we visualized and quantified these factors which may provide a predictive tool for new treatments. Twenty-one patients with stage III–IV squamous cell carcinomas of the head and neck were intravenously injected with pimonidazole, a bioreductive hypoxic marker. Tumour biopsies were taken 2 h later. Frozen tissue sections were stained for vessels and hypoxia by fluorescent immunohistochemistry. Twenty-two sections of biopsies of different head and neck sites were scanned and analysed with a computerized image analysis system. The hypoxic fractions varied from 0.02 to 0.29 and were independent from T- and N-classification, localization and differentiation grade. No significant correlation between hypoxic fraction and vascular density was observed. As a first attempt to categorize tumours based on their hypoxic profile, three different hypoxia patterns are described. The first category comprised tumours with large hypoxic, but viable, areas at distances even greater than 200 μm from the vessels. The second category showed a typical band-like distribution of hypoxia at an intermediate distance (50–200 μm) from the vessels with necrosis at greater distances. The third category demonstrated hypoxia already within 50 μm from the vessels, suggestive for acute hypoxia. This method of multiparameter analysis proved to be clinically feasible. The information on architectural patterns and the differences that exist between tumours can improve our understanding of the tumour micro-environment and may in the future be of assistance with the selection of (oxygenation modifying) treatment strategies. © 2000 Cancer Research Campaign


*British Journal of Cancer* (2000) **83**(5), 674–683
© 2000 Cancer Research Campaign
doi: 10.1054/ bjoc.2000.1325, available online at http://www.idealibrary.com on IDEAL®

### Vascular architecture and hypoxic profiles in human head and neck squamous cell carcinomas

**KIEM Wijffels[1,2], JHAM Kaanders[1], PFJW Rijken[1], J Bussink[1], FJA van den Hoogen[2], HAM Marres[2], PCM de Wilde[3], JA Raleigh[4] and AJ van der Kogel[1]**

[1]Institute of Radiotherapy, [2]Department of Otorhinolaryngology, [3]Departments of Pathology and Maxillofacial Surgery, University of Nijmegen, P.O. Box 9101, 6500 HB Nijmegen, The Netherlands; [4]Radiation Oncology and Toxicology, UNC School of Medicine, Chapel Hill, USA

**Summary** Tumour oxygenation and vasculature are determinants for radiation treatment outcome and prognosis in patients with squamous cell carcinomas of the head and neck. In this study we visualized and quantified these factors which may provide a predictive tool for new treatments. Twenty-one patients with stage III–IV squamous cell carcinomas of the head and neck were intravenously injected with pimonidazole, a bioreductive hypoxic marker. Tumour biopsies were taken 2 h later. Frozen tissue sections were stained for vessels and hypoxia by fluorescent immunohistochemistry. Twenty-two sections of biopsies of different head and neck sites were scanned and analysed with a computerized image analysis system. The hypoxic fractions varied from 0.02 to 0.29 and were independent from T- and N-classification, localization and differentiation grade. No significant correlation between hypoxic fraction and vascular density was observed. As a first attempt to categorize tumours based on their hypoxic profile, three different hypoxia patterns are described. The first category comprised tumours with large hypoxic, but viable, areas at distances even greater than 200 μm from the vessels. The second category showed a typical band-like distribution of hypoxia at an intermediate distance (50–200 μm) from the vessels with necrosis at greater distances. The third category demonstrated hypoxia already within 50 μm from the vessels, suggestive for acute hypoxia. This method of multiparameter analysis proved to be clinically feasible. The information on architectural patterns and the differences that exist between tumours can improve our understanding of the tumour micro-environment and may in the future be of assistance with the selection of (oxygenation modifying) treatment strategies. © 2000 Cancer Research Campaign

**Keywords:** pimonidazole; hypoxia; vascular density; head and neck carcinomas

The relevance of blood flow for the outcome of radiation treatments was already described by Schwartz in 1909. He noticed that skin reactions were less severe when the radium applicator was firmly pressed to the skin. It was Cramer in 1935 who gave the first suggestion of an ‘oxygen effect’. He found that mouse mammary tumours with a well developed stroma and vascular network were more radiosensitive than tumours with a more delicate stromal component. He suggested that the radiosensitive tumours had a better blood supply and, consequently, were better oxygenated. Later, Gray et al (1953) established the importance of tumour oxygenation for radiotherapy.

The tumour micro-environment is a key factor in tumour biology and has great impact on clinical radiotherapy as well as other cancer treatments. Single parameters such as polarographic $pO_2$ measurements and microvascular density have been demonstrated to be prognostic indicators in a variety of tumour types, including carcinomas of the head and neck (Gatenby et al, 1987; Dray et al, 1995; Zätterström et al, 1995; Jenssen et al, 1996; Nordsmark et al, 1996; Brizel et al, 1997). However, considering the complexity of the micro-environmental system, it is unlikely that a single parameter can reliably predict treatment outcome on an individual basis. Multiple parameter analysis will be necessary to acquire a better understanding of the tumour micro-environment and to obtain a ‘predictive profile’ which can guide the clinician in the selection of patients for new treatment strategies.

A computerized image analysis system for multi-parameter analysis has been developed in our institute (Rijken et al, 1995). The method involves computer controlled microscopic scanning of immunohistochemically stained tissue sections. It allows quantitative and simultaneous analysis of vascular parameters, oxygenation status and proliferation parameters. We have recently published our experience with this method in xenografted human tumours (Bussink et al, 1998, 1999).

We now apply this method also to tumour biopsies from patients with squamous cell carcinomas of the head and neck. Before a biopsy was taken, the patients were injected with Hypoxyprobe-1 (pimonidazole hydrochloride) as a hypoxia marker. Pimonidazole can easily be dissolved in saline, has a low toxicity, and an efficient tumour uptake. So far pimonidazole has proven to be an effective hypoxia marker for human tumours of the cervix and head and neck (Kennedy et al, 1997; Raleigh et al, 1998; Varia et al, 1998). In the present study we report the results of the analysis of biopsy material from 21 patients with squamous cell carcinoma of the head and neck.

*Received 19 January 2000*
*Revised 30 March 2000*
*Accepted 3 April 2000*

*Correspondence to:* KIEM Wijffels

### MATERIALS AND METHODS

### Patients

Patients with primary laryngeal, oropharyngeal (stage III–IV) or hypopharyngeal (stage II–IV) squamous cell carcinomas were entered. Accrual was restricted to patients who were also eligible for our ongoing phase II ARCON-study (Accelerated Radiotherapy with Carbogen and Nicotinamide) (Kaanders et al, 1995). Inclusion criteria were: age over 18 years, WHO performance status of 0–2, no severe heart or lung disease, no severe liver or kidney dysfunction, no severe stridor, no distant metastases, and written informed consent. In the period from May 1998 to December 1998, 21 patients were included. Approval from the local ethics committee was obtained.

### Markers of hypoxia and proliferation

As hypoxia marker we used pimonidazole hydrochloride (Hypoxyprobe-1, Natural Pharmacia International Inc). Pimonidazole is a bioreductive chemical probe with an immunorecognizable side chain. The addition of the first electron in bioreductive activation is reversibly inhibited by oxygen with a half maximal $pO_2$ of inhibition of 3 mmHg and almost complete inhibition at about 10 mmHg (Raleigh et al, 1999). Pimonidazole (0.5 g/m$^2$) was dissolved in 100 cc NaCl 0.9% and was administered intravenously over 20 minutes, two hours before biopsy (range 1 h 35 min–4 h 05 min, median 1 h 59 min). A maximum dose of one gram was given to patients with a body surface >2 m$^2$. After biopsy the tissue was directly frozen in liquid nitrogen. Sections of 5 μm were cut and mounted on poly-L-lysine coated slides and stored at –80°C until staining.

### Immunohistochemical staining

Prior to the staining procedure, sections were fixed in acetone of 4°C for 10 min. Then the sections were rehydrated in phosphate buffered saline (PBS). Next, sections were incubated overnight at 4°C with both anti-pimonidazole (rabbit polyclonal) (Azuma et al, 1997) diluted 1:200 in PBS and PAL-E diluted 1:5 in PBS (Department of Pathology, University Hospital Nijmegen, The Netherlands). The monoclonal antibody PAL-E is a marker for human endothelium, especially useful in frozen tissue sections (Schlingemann et al, 1985). After rinsing three times in PBS the staining procedure was followed by a pooled incubation for 1 h at room temperature with fluorescein isothiocyanate (FITC)-conjugated donkey anti-rabbit antibody (Jackson Immuno Research Laboratories, West Grove, PA, USA) 1:100 in PBS for the hypoxia signal, combined with tetramethyl rhodamine isothiocyanate (TRITC)-conjugated goat anti-mouse antibody (Jackson Immuno Research Laboratories, West Grove, PA, USA) diluted 1:100 in PBS to visualise the vessels. After rinsing in PBS for three times the sections had another pooled incubation for 1 h with the same FITC-conjugated antibody, but now combined with TRITC-conjugated donkey anti-goat antibody (Jackson Immuno Research Laboratories) diluted 1:100 in PBS to amplify the blood vessel signal. Slides were then rinsed and mounted in PBS for scanning of the hypoxia and vessel signals.

### Scanning of tumour sections and image processing

For quantitative analysis, the slides were scanned by a computerized image processing system using a high-resolution intensified solid-state video camera on a fluorescence microscope (Zeiss Axioskop). The fluorescence signals were recorded by the camera and subsequently digitized to a binary image on a Macintosh computer. For the vessels (TRITC-signal, 510–560 nm excitation) a 590 nm emission filter was used and for hypoxia (FITC-signal, 450–490 nm excitation) a 520 nm emission filter was used. In order to move a tissue section automatically a motorized scanning stage, coupled to a stage controller, was interfaced with the computer. Each tumour section was scanned two times at 100× magnification. In the first scan only the vascular structures were detected, in the second scan the hypoxic marker was detected. Each scan consisted of 16, 25 or 36 fields (4×4, 5×5 or 6×6, field size 1.22 mm$^2$) depending on the size of the biopsy. Processing all fields of one scanning procedure resulted in a composite binary image. After the scanning procedure the two composite binary images, one showing vascular structures and the other showing hypoxic areas, were superimposed. A detailed description of the scanning method has been given by Rijken et al (1995). With the use of a haematoxylin-eosin staining of a consecutive section the tumour area was delineated. This area was used as a mask in further image analysis excluding non-tumour tissue and large necrotic areas from the analysis. Necrosis can produce an aspecific red stain in the fluorescent images. It is therefore important that these areas be excluded such that they do not disturb the analysis of vascular parameters. Small necrotic areas were not excluded because this could cause loss of information of the directly surrounding viable tissue. The delineation of the tumour area was supervised by a pathologist (PCM de Wilde).

### Analysis of hypoxic and vascular parameters

The vascular density was calculated as the number of vascular structures per mm$^2$. The relative vascular volume was defined as the PAL-E positive surface divided by the total tumour surface. The hypoxic fraction was defined as the pimonidazole positive surface divided by the total tumour surface. These parameters were calculated for the complete tumour surface, but also in arbitrary subareas of 0.3 mm$^2$ for analysis of the intra-tumour variability.

In order to quantitate the distribution of hypoxia in relation to the vasculature, zones were chosen arbitrarily at increasing distance from the surface of the nearest vessel (0–50 μm, 50–100 μm, 100–150 μm, 150–200 μm, 200–250 μm, and >250 μm). The hypoxic fraction in a zone was calculated as the pimonidazole-stained surface in a zone divided by the tumour surface in that zone.

### Statistics

Mean values were compared by the student-t-test. Correlations between various parameters were tested by linear least-squares regression analysis. The statistical analyses were done on a Macintosh computer using Statistica 4.0 software.

### RESULTS

### Patients

Twenty-three patients entered the study. Two biopsies were not included in the analysis, one biopsy contained no tumour tissue and one patient had a mucoepidermoid carcinoma. Thus biopsies from 21 patients with confirmed squamous cell carcinoma were the subject of this study. The median age of the patients was 58

© 2000 Cancer Research Campaign

**Table 1** TNM-classification of the 22 biopsied tumours

| | N0 | N1 | N2 | N3 | Total |
|---|---|---|---|---|---|
| T1 | – | 1 | 1 | – | 2 |
| T2 | 1 | 1 | 3[a] | 2 | 7 |
| T3 | 2 | 1 | 5[a] | – | 8 |
| T4 | – | 2 | 3 | – | 5 |
| Total | 3 | 5 | 12 | 2 | 22 |

[a] Biopsies from one patient with two primary tumours years (range 47–75). There were 17 men and 4 women. Tumours were localized in the oropharynx (2), in the larynx (9) and in the hypopharynx (9). One patient suffered from two primary tumours, an oropharyngeal and a hypopharyngeal tumour which were both biopsied. Thirteen tumours were moderately differentiated and nine were poorly differentiated. Classification of the tumours according to the TNM staging system (as defined by the UICC, 1997) is shown in Table 1. None of the patients had any adverse reaction to the pimonidazole administration.

### Hypoxia and vessel staining

The hypoxic staining gave a reproducible and bright green fluorescent signal in all tissue sections. At increasing distance from the vessels an increasing intensity of the hypoxic staining was found. There was no or very little background staining (Figure 1). At the edges of some tumour sections we observed a strong staining. This so called ‘edge-effect’ was sometimes substantial and, if included in the analysis, could influence the outcome significantly. Three different causative phenomena were identified. First, freezing artefacts occur sometimes at the tissue edges which are caused by the formation of ice crystals with subsequent tissue damage. These freezing artefacts are known to give non-specific staining with immunohistochemical techniques. Secondly, the edges of tissue sections can become pleated while being cut, which enhances the background staining. The third reason is the presence of normal epithelium covering the surface of a biopsy. The differentiated squamous layers can demonstrate considerable hypoxia. The latter is obviously not an artefact but was excluded from the analysis as non-tumour tissue. In the majority of the biopsies no or very little normal epithelium was identified.

The red fluorescent vessel staining gave a strong signal in all tumours with hardly any background staining. Some aspecific staining was observed in areas of necrosis, which also can contain remnants of blood vessels (Figure 1).

### Staining patterns

Based on the vascular architecture and the pattern of hypoxic staining we have tried to categorize the tumours into three groups. Category A ($n$ = 8) showed increasing amounts of hypoxia with increasing distance from the vessels and large areas of hypoxia at distances greater than 200 μm. They had almost no hypoxia close to the vessels (Fig. 2A). Category B ($n$ = 4) tumours had a typical

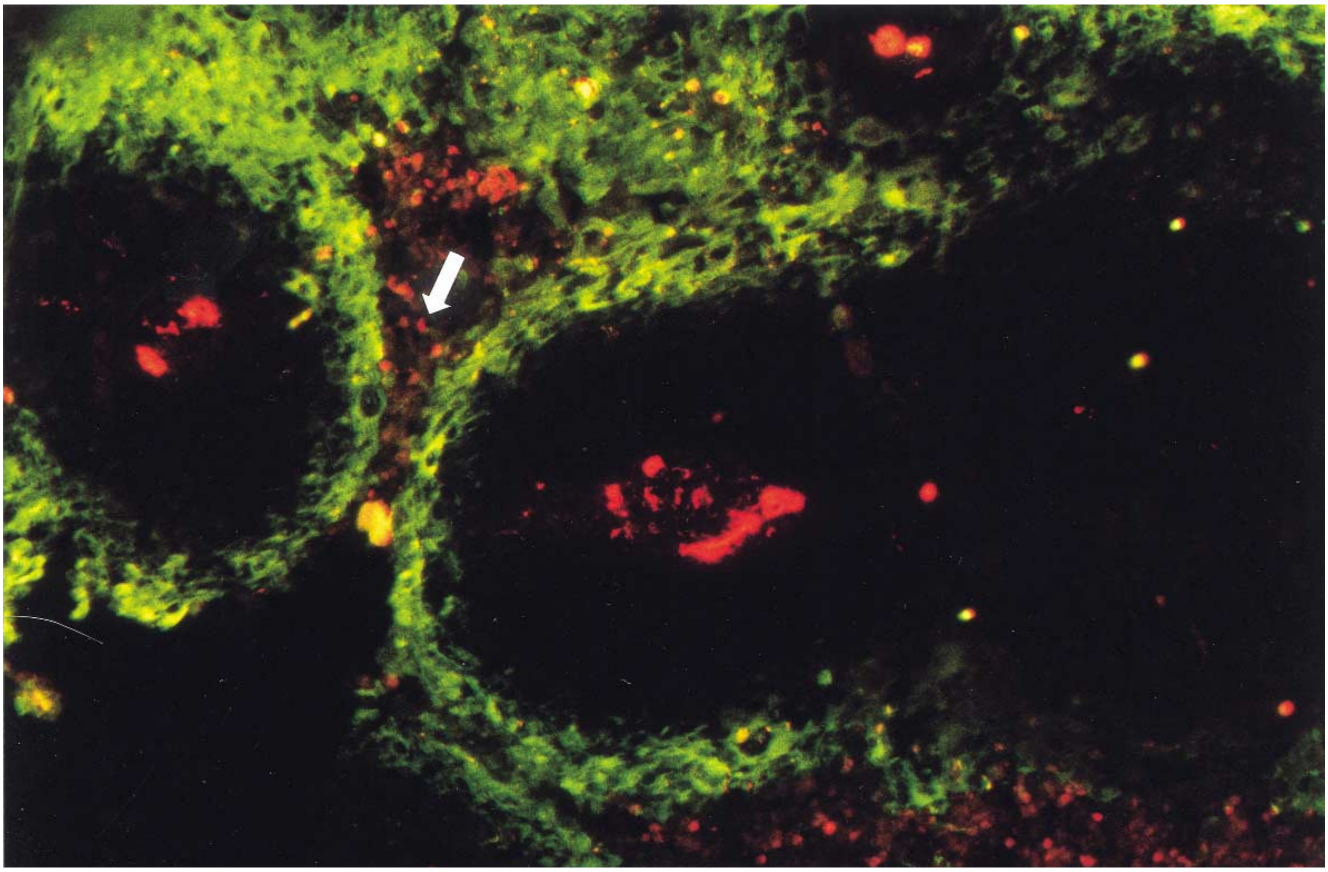

**Figure 1** Fluorescence microscopic image of a tumour section (200 × magnification) after staining for hypoxia with pimonidazole (green) and vessels with PAL-E (red). Typical tumour cords can be recognized with well oxygenated areas directly adjacent to vessels and hypoxia at greater distance. At even greater distances there is necrosis (white arrow, confirmed in H&E stain)

 *© 2000 Cancer Research Campaign*

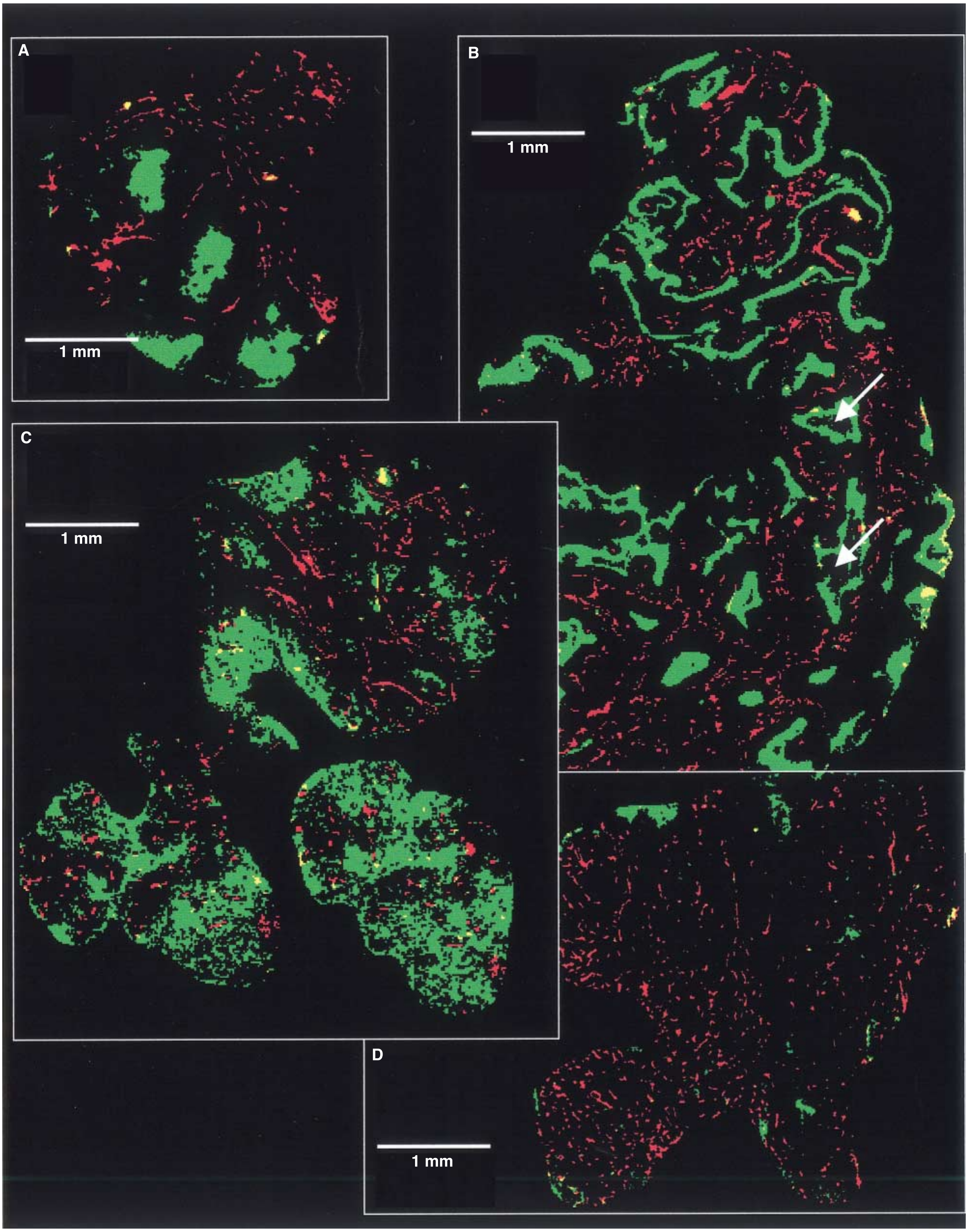

**Figure 2** Composite binary images of four different tumours; sccNij50 (A), sccNij70 (B), sccNij49 (C) and sccNij69 (D) showing hypoxia (green) and vessels (red). The tumours represent examples of different hypoxic patterns. Large necrotic areas have been excluded from these binary images. Only in tumour sccNij70 there were areas of necrosis that were too small to be excluded (arrows)

band-like pattern of hypoxia at shorter distances from the vessels (between 50 and 200 μm) (Fig. 2B). In this category of tumours we found small areas of necrosis at distances >150–200 μm. This was confirmed in the haematoxylin-eosin staining but these areas were too small to be excluded from the analysis. Category C demonstrated a more diffuse hypoxic pattern with significant amounts of hypoxia in the zones closest to the vessels. The relation to the vasculature was less obvious in this category ($n$ = 4) (Fig. 2C). Even tumours with similar hypoxic fractions could vary in their hypoxic patterns. Six tumours had small hypoxic fractions of less than 5% and, therefore, could not be properly classified (Fig. 2D).

### Quantitative analysis

### *Vascular parameters*

The vascular density (VD) of the 22 tumours ranged from 11 $mm^{-2}$ to 67 $mm^{-2}$ with a median value of 42 $mm^{-2}$. The vascular density was also calculated in arbitrary subareas of 0.3 $mm^2$. The highest values of these subareas were a factor of 1.5 to 6 higher than the overall values of the various tumours.

The relative vascular volume varied from 1.9% to 9.0%, with a median of 3.8%. There was no correlation between the vascular parameters and tumour site, T- and N-stage or histological grade.

### *Hypoxia*

Figure 3 presents the hypoxic fractions of the tumours as a cumulative plot. The lowest and highest values were 0.02 and 0.29, respectively, with a median of 0.09. There was an indication that pharyngeal tumours had a higher hypoxic fraction (median 0.10) as compared to the larynx tumours (median 0.05). However, these differences were not statistically significant ($P$ = 0.21, t-test). There was also no correlation between T- and N-stage or histological grade and the hypoxic fraction.

Hypoxic fractions were also calculated for the subareas in each tumour and cumulative plots were constructed. Figure 4 shows these plots for the three tumours of which the corresponding images are shown in Figure 2. The steepest curve with the lowest median value (=0.03) corresponds with the tumour that demonstrates hypoxia mainly at large distances from vessels (category A). The plot for the tumour with the more diffuse hypoxic pattern (category C) is less steep with a much higher median value (=0.26).

### *Relation between vasculature and hypoxia*

We sought for further correlations between vascular density, relative vascular volume, and hypoxic fraction. There was a significant correlation between relative vascular volume and vascular density (r = 0.56, $P$ < 0.01). There was only a weak and non-significant inverse correlation between vascular density and hypoxic fraction (r = −0.34, $P$ = 0.13) (Figure 5).

In all tumours there was an increase of hypoxia with increasing distance from the vessels. As an example, Figure 6 shows how the arbitrary zones chosen at increasing distances from the vessels are projected over the binary image of vessels and hypoxia in tumour sccNij50. Figure 7 shows the relative distribution of hypoxia over these zones for the tumours of Figure 2A–C.

### DISCUSSION

### Methods

With the quantitative analysis method used here we successfully visualized and quantitated multiple parameters while maintaining

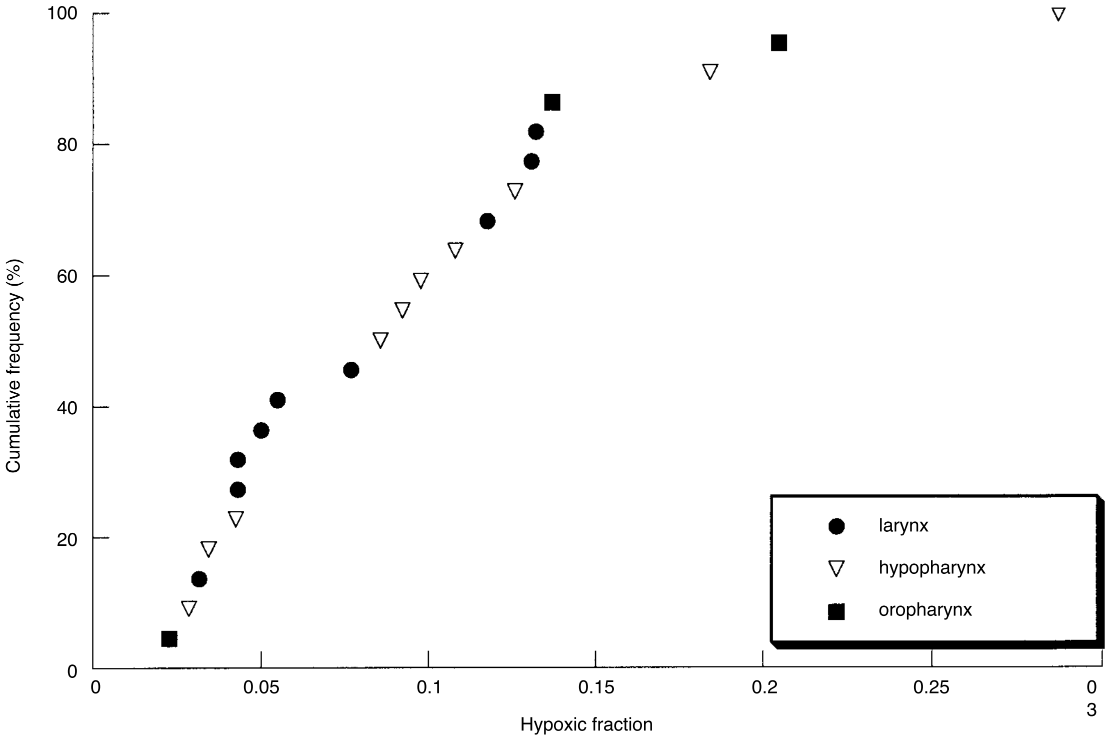

**Figure 3** Cumulative plot of hypoxic fractions of 22 tumours originating in three head and neck sites

 *© 2000 Cancer Research Campaign*

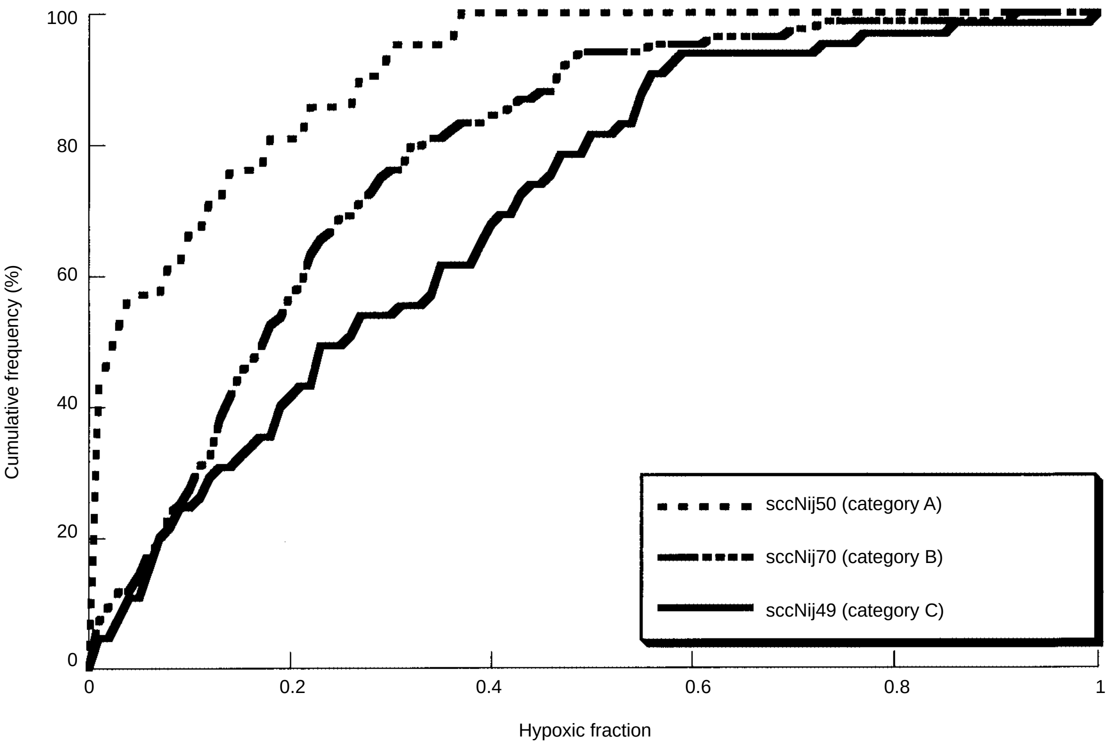

**Figure 4** Cumulative plot of the hypoxic fractions of arbitrary subareas in three tumours (sccNij50, sccNij70, sccNij49)

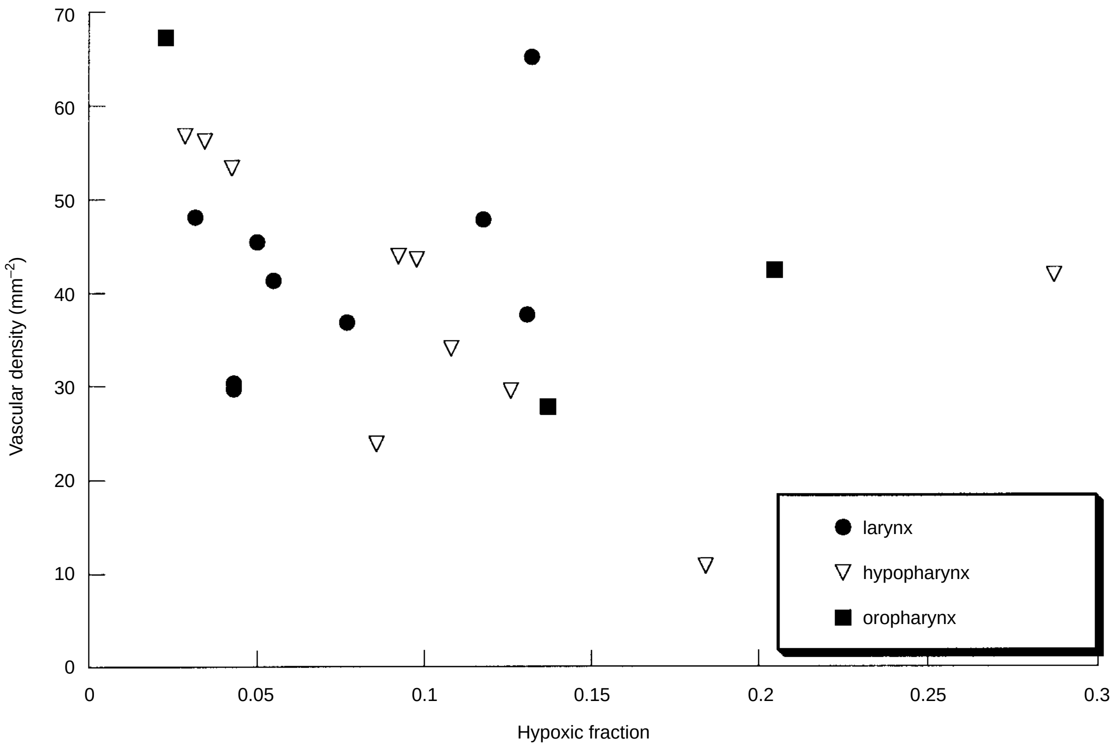

**Figure 5** Vascular density versus hypoxic fraction in 22 tumours of three head and neck sites the tissue architecture (Rijken et al, 1995; Bussink et al, 1998, 1999). To obtain a strong signal with lower background staining we used fluorescent labelled antibodies on frozen sections. A disadvantage of the immunohistochemical staining is that only specific structures are stained, i.e. the complete tissue architecture is not visualized. Therefore we used haematoxylin-eosin staining of consecutive sections to contour the tumour area, excluding non-tumour tissue and areas of necrosis. Both the hypoxia and the

*© 2000 Cancer Research Campaign*

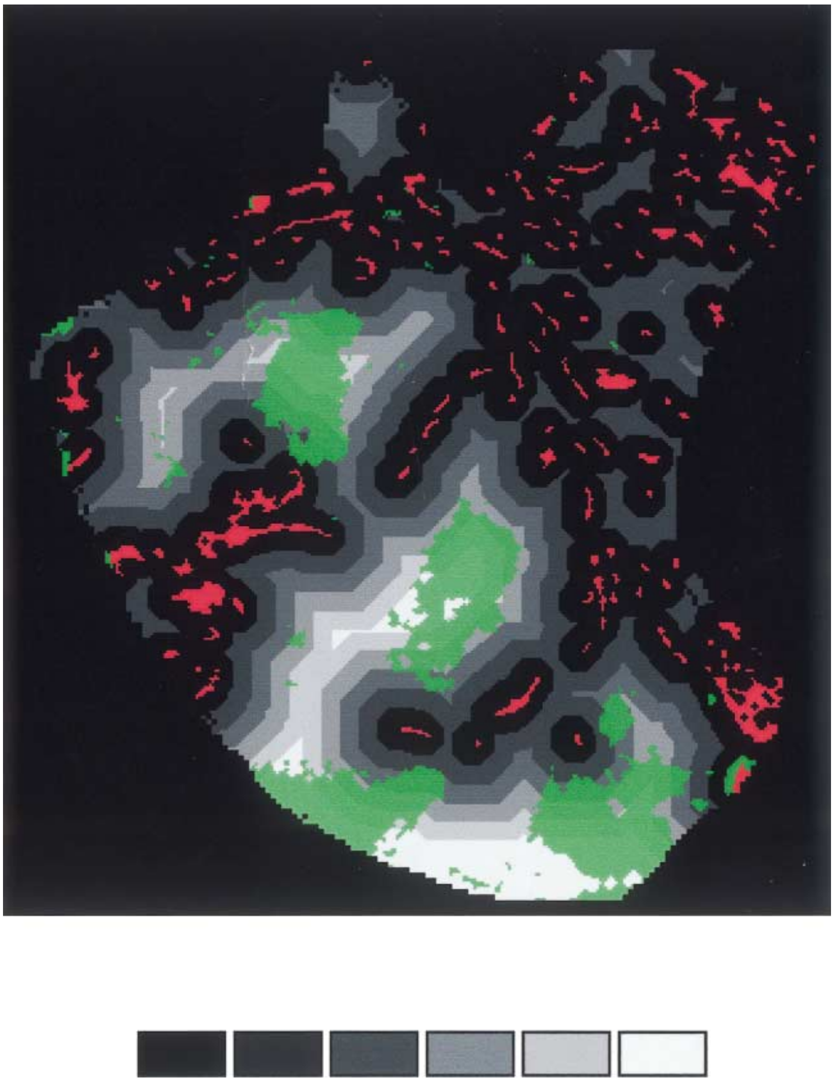

**Figure 6** Binary image of vessels (red) and hypoxia (green) in tumour sccNij50 with the zones at increasing distances from the vessels superimposed vessel stainings gave strong signals with very low background staining in all slides. Edge artefacts were easily recognized with the aid of the haematoxylin-eosin stained sections and were excluded from the analysis.

### Vascular parameters

There is an abundance of literature on morphometric vascular tumour parameters, in particular microvascular density, and their prognostic value. For squamous cells carcinomas of the head and neck, as for other tumour types such as carcinoma of the breast, conflicting results have been reported. Some investigators found a correlation between vascular density and metastatic potential (Gasparini et al, 1993; Albo et al, 1994; Shpitzer et al, 1996; Murray et al, 1997), whereas others did not (Leedy et al, 1994; Dray et al, 1995; Zätterström et al, 1995; Janot et al, 1996; Moriyama et al, 1997; Salven et al, 1997; Burian et al, 1999). In this study no correlation was found between vascular density and N-stage, even when the 75th percentile value or the highest value of the subareas were used. The latter analysis was done to approximate the so called ‘hot spot counting’ method which is often used by others (Gasparini et al, 1993; Leedy et al, 1994; Shpitzer et al, 1996; Murray et al, 1997; Salven et al, 1997). Two investigators found an association between vascular density and local tumour control after radiation treatment (Jenssen et al, 1996; Aebersold et al, 1999). Unfortunately, the relationship was opposite in the two studies, one study (Jenssen et al, 1996) demonstrating increased risk of local recurrence with low vascular density but the other (Aebersold et al, 1999) with high vascular density. The current study is being continued and patient numbers and follow-up will be increased to allow meaningful correlations between the micro-environmental parameters and treatment outcome.

Absolute values of vascular density are difficult to compare between studies, mainly because of methodological differences, in particular the type of endothelial marker used and the counting technique. Our values are in the lower range compared with other studies. This can be explained by the fact that in most studies vessels were counted in selected, highly vascularized areas

 © 2000 Cancer Research Campaign

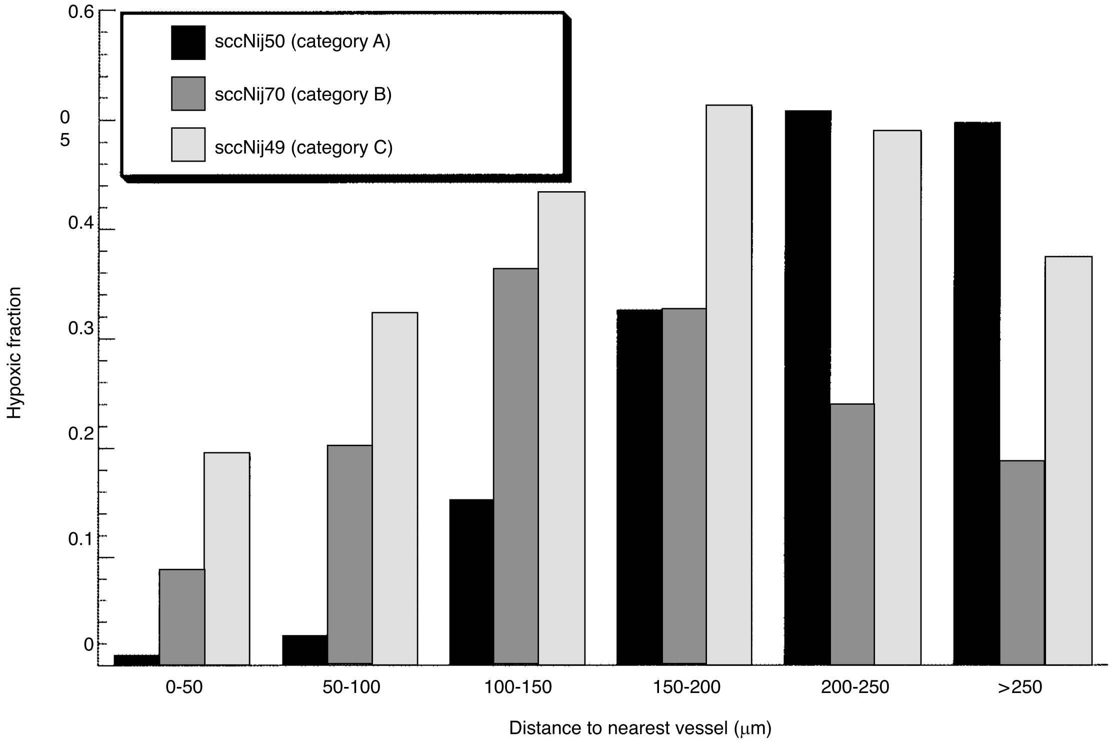

**Figure 7** Relative hypoxic fractions in 50 μm zones at increasing distance from vessels. Example of three tumours (sccNij50, sccNij70, sccNij49)

('hotspots'), whereas in the present analysis figures are based on the counting of an entire biopsy section. Indeed, the highest values obtained from the arbitrary subareas correspond better with values reported in literature. We found only a weak and non-significant correlation between the overall vascular density and hypoxic fraction. This indicates that a global assessment of vascularity is probably not a good parameter for tumour oxygenation, due to the heterogeneity in vasculature and oxygenation in tumours. Lyng et al (1997) also demonstrated that there are large regional differences in vascularity and oxygenation in carcinomas of the uterine cervix. At the microregional level they did however observe a correlation between low $pO_2$ readings by the polarographic method (Eppendorf Histograph) and vascularity.

### Hypoxia

Various methods have been described to measure tumour tissue oxygenation including polarographic oxygen electrodes, magnetic resonance spectroscopy, haemoglobin saturation assays, radionuclide assays, and, as used in the present study, by immunohistochemical markers. Methods for assessing the radiobiological hypoxic fraction include the classical paired survival curve assay and, more recently, the comet assay (Olive et al, 1998). Most clinical data have been obtained with the oxygen micro-electrodes. The low water solubility of the earlier immunohistochemical hypoxia markers prohibited their introduction in the clinic. Pimonidazole hydrochloride however has a high water solubility and can be easily administered intravenously in a small volume of aqueous solution. It distributes rapidly into the tissues where concentrations threefold of that in plasma are reached. These properties and the absence of any significant adverse effects with the amount required make this agent attractive for clinical use.

Raleigh et al (1999) compared pimonidazole binding with micro-electrode measurements and with measurements of the radiobiologically hypoxic fraction in the C3H mouse mammary carcinoma. The oxygenation of these tumours was manipulated by the breathing of air, hyperoxic gases, injection of hydralazine and tumour clamping. They showed that pimonidazole binding was well correlated with both micro-electrode measurements and the radiobiological hypoxic fraction. A good correlation was found between pimonidazole binding and percentage of micro-electrode measurements ≤10 mmHg. This is consistent with the intracellular binding properties of pimonidazole, rising steeply at $pO_2 < 10$ mmHg. However, it was also clear from their data that the micro-electrodes systematically overestimate the extent of hypoxia at the cellular level. Under well-oxygenated circumstances where pimonidazole binding was almost negligible, the micro-electrodes still recorded a significant number of readings ≤10 mmHg (36%). One of the explanations given was the presence of necrosis which produces low readings with the micro-electrodes while there is no pimonidazole binding in these areas.

Also, when our results of hypoxic fraction assessed by pimonidazole binding (median 0.09) are compared with studies of micro-electrode measurements in head and neck cancer patients, the latter report substantially higher values (Gatenby et al, 1987; Nordsmark et al, 1996; Brizel et al, 1997). In these studies the median of the fraction of $pO_2$ readings <5 mmHg varied from 0.29 to 0.41. The fraction of $pO_2$ values < 10 mmHg was generally not reported. These measurements were mostly taken from metastatic neck nodes in contrast to our biopsy material which originated from the primary tumours in all cases. Central necrosis is often observed in cross-sectional CT imaging of nodal metastasis, and

*© 2000 Cancer Research Campaign* 

this may be one explanation for the discrepancy between our results and those of the polarographic studies.

Initial studies on the clinical use of pimonidazole as a hypoxia marker came from the group from the University of North Carolina (Kennedy et al, 1997; Raleigh et al, 1998; Varia et al, 1998). Raleigh et al (1998) reported on 18 patients with carcinomas of the uterine cervix and head and neck. The area fraction labelled with pimonidazole varied from almost zero to approximately 0.25 with a median of 0.02. Varia et al (1998) reported on 10 patients with carcinoma of the uterine cervix. Values for pimonidazole binding were below 0.1 in 9 of the 10 patients. It is difficult to compare the results from these two studies with the current study because of the differences in tumour sites and the relatively small sample sizes. Also, different methodologies were used for assessment of the hypoxic fraction.

Another bioreductive compound of the nitroimidazole group is 'EF5', a pentafluorinated derivate of etanidazole. This recently developed immunohistochemically detectable hypoxic marker has been tested in animal tumours and xenografted human tumours (Fenton et al, 1999). Very recently, Evans et al (2000) reported the first clinical application of this marker.

### Patterns of hypoxia

As demonstrated various patterns of hypoxia can be recognized (Figure 2). Differences between these patterns can be quantified by calculating the hypoxic fractions of arbitrary subareas (Figure 4) and, maybe more interesting, by quantifying hypoxia as a function of distance from the vessels (Figures 6 and 7). The categorization of the tumours was based on the visual aspect of the hypoxia staining combined with the quantitative analysis of the hypoxia distribution over the vascular zones. The tumours shown in Figures 1 and 2b are typical examples of the classical 'cord-like' tumour model described by Thomlinson and Gray (1955). Diffusion limited hypoxia was the predominant feature of all the tumours we analysed. However, the spatial relationship to the vessels was not always the same. In the tumour of category A hypoxia was virtually absent within 100 μm of vascular structures. Hypoxia was predominantly present at large (150 μm → 250 μm) distances from the vessels. In this tumour all necrotic areas could be excluded from the analysis, i.e. Figures 2a and 6 depict only viable tumour tissue. Thus, significant amounts of viable tumour tissue, both hypoxic and non-hypoxic (e.g. six o'clock position in Figure 6) were observed at large (>250 μm) distances from blood vessels. It can, however, not be excluded that these areas might have had oxygen and nutritional supply from vessels above or below the section examined. The analysis system used provides only a two-dimensional image of the tumour which obviously is a limitation. On the other hand, there is no good reason to assume that the vascular architecture will be any different in the third dimension. Since this category A tumour demonstrates large intervascular distances, it is most likely that vascular structures are sparse, also above and below the plane of the section analysed. The category B tumour showed already some hypoxia in the first 50 μm zone (hypoxic fraction 0.09) and a gradual increase of the hypoxic fraction in the second and the third zones. However, at distances > 150 μm hypoxia seemed to decrease again. The explanation for this unexpected phenomenon is a 'contamination' by small areas of necrosis at these greater distances (see arrows in Figure 2B). This was confirmed in the haematoxylin-eosin staining. These necrotic areas were too small however to be excluded from the analysis and because they were not labelled by pimonidazole, in the computer calculations they were falsely considered as 'viable non-hypoxic tumour tissue'. Thus, in this particular tumour, the hypoxic fractions at distances >150 μm were underestimated. Nevertheless, it is an interesting observation that, while in one category of tumours (A) there are significant amounts of viable tissue at distances >250 μm, in other tumours there is already necrosis at 150–200 μm (category B). These differences suggest that the balance between oxygen delivery and oxygen consumption may vary among tumours of the same histology resulting in different distributions of chronic hypoxia and necrosis.

The tumour of category C demonstrated a considerable amount of hypoxia within 100 μm from the vessels with a hypoxic fraction of 0.20 at 0–50 μm. This suggests impaired functioning of particular vessels, either permanently or temporarily ('acute hypoxia'). This may also be the case, but to a lesser extent, in the category B tumour. In xenografted tumours we demonstrated that after 10 min of exposure to pimonidazole there is a weak binding in the hypoxic cells which increases between 10 and 30 min of exposure (unpublished data). This indicates that, with this assay, acute hypoxia probably will only be recognized if cells are hypoxic for at least 10 min.

Together with single parameters such as vascular density and hypoxic fraction, the pattern of hypoxic staining may give additional prognostic information and may help to select appropriate treatment modalities. We have previously demonstrated that carbogen can very efficiently reduce diffusion limited hypoxia in human squamous cell carcinoma xenografts (Bussink et al, 1999). However, preliminary data from our laboratory suggest that it is difficult to eradicate all the hypoxia at greater distance from the vessels. This may also be the case in the category A tumours. At the same time there seems to be limited value in adding vasoactive agents such as nicotinamide to the treatment of such tumours since they exhibit no or only very little acute hypoxia. Our attempt to categorise human tumours based on hypoxic patterns must be validated with a larger number of patients. This is the subject of ongoing investigations.

### CONCLUSION

We have presented a quantitative method for multiparameter analysis of vascularity and hypoxia in head and neck tumours which is clinically feasible. These measurements can be of prognostic relevance. Moreover, this method yields information on architectural patterns which may improve our understanding of the tumour micro-environment and may in the future be of assistance with the selection of treatment strategies.

### ACKNOWLEDGEMENTS

The authors thank Professor J Denekamp for her advice on the design of this study and the analysis of the results. Also the authors would like to thank JPW Peters for expert technical assistance. We would like to thank the Dutch cancer society for the financial support of this study.

### REFERENCES

Aebersold DM, Beer KT, Laissue J, Greiner RH and Djonov V (1999) Intratumoral microvessel density predicts local failure of radically irradiated squamous cell cancer of the oropharynx. *Eur J Cancer* **35** (Suppl 4): 179

© 2000 Cancer Research Campaign

Albo D, Granick MS, Jhala N, Atkinson B and Solomon MP (1994) The relationship of angiogenesis to biological activity in human squamous cell carcinomas of the head and neck. *Ann Plast Surg* **32**: 588–594

Azuma C, Raleigh JA and Thrall E (1997) Longevity of pimonidazole adducts in spontaneous canine tumours as an estimate of hypoxic cell lifetime. *Radiation Research* **148**: 35–42

Brizel DM, Sibley GS, Prosnitz LR, Scher RL and Dewhirst MW (1997) Tumor hypoxia adversely affects the prognosis of carcinoma of the head and neck. *Int J Radiat Oncol Biol Phys* **38**: 285–289

Burian M, Quint CH and Neuchrist C (1999) Angiogenic factors in laryngeal carcinomas: Do they have prognostic relevance? *Acta Otolaryngol* **119**: 289–292

Bussink J, Kaanders JHAM, Rijken PFJW, Martindale CA and Van der Kogel AJ (1998) Multiparameter analysis of vasculature, perfusion and proliferation in human tumor xenografts. *Br J Cancer* **77**: 57–64

Bussink J, Kaanders JHAM, Rijken PFJW, Peters JPW, Hodgkiss RJ, Marres HAM and Van der Kogel AJ (1999) Vascular architecture and microenvironmental parameters in human squamous cell carcinoma xenografts: effects of carbogen and nicotinamide. *Radiother Oncol* **50**: 173–184

Cramer W (1935) The therapeutic action of radium on spontaneous mammary carcinomata of the mouse. *Annual report of the Imperial Cancer Research Fund Report* **11**: 127–146

Dray TG, Hardin NJ and Sofferman RA (1995) Angiogenesis as a prognostic marker in early head and neck cancer. *Ann Otol Rhinol Laryngol* **104**: 724–729

Evans SM, Hahn S, Pook DR, Jenkins WT, Chalian AA, Zhang P, Stevens C, Weber R, Weinstein G, Benjamin I, Mirza N, Morgan M, Rubin S, McKenna WG, Lord EM and Koch CJ (2000) Detection of hypoxia in human squamous cell carcinoma by EF5 binding. *Cancer Research* in press

Fenton BM, Paoni SF, Lee J, Koch CJ and Lord EM (1999) Quantification of tumor vasculature and hypoxia by immunohistochemical staining and HbO2 saturation measurements. *Br J Cancer* **79**: 464–471

Gasparini G, Weidner N, Maluta S, Pozza F, Boracchi P, Mezzetti M, Testolin A and Bevilaqua P (1993) Intratumoral microvessel density and P53 protein: correlation with metastasis in head-and-neck squamous-cell carcinoma. *Int J Cancer* **55**: 739–744

Gatenby RA, Kessler HB, Rosenblum JS, Coia LR, Moldofsky PJ, Hartz WH and Broder GJ (1987) Oxygen distribution in squamous cell carcinoma metastases and its relationship to outcome of radiation therapy. *Int J Radiat Oncol Biol Phys* **14**: 831–838

Gray LH, Conger AD, Ebert M, Hornsey S and Scott OCA (1953) The concentration of oxygen dissolved in tissues at the time of irradiation as a factor in radiotherapy. *Br J Radiol* **26**: 638–648

International Union Against Cancer (1997) *TNM classification of malignant tumours*. 5th edition. Wiley-Liss: New York

Janot F, Klijanienko J, Russo A, Mamet J-P, de Braud F, El-Naggar AK, Pignon J-P, Luboinski B and Cvitkovic E (1996) Prognostic value of clinicopathological parameters in head and neck squamous cell carcinoma: a prospective analysis. *Br J Cancer* **73**: 531–538

Jenssen N, Boysen M, Kjærheim Å and Bryne M (1996) Low vascular density indicates poor response to radiotherapy in small glottic carcinomas. *Path Res Pract* **192**: 1090–1094

Kaanders JHAM, Pop LAM, Marres HAM, van der Maazen RWM, van der Kogel AJ and van Daal WAJ (1995) Radiotherapy with carbogen breathing and nicotinamide in head and neck cancer: feasibility and toxicity. *Radiother Oncol* **37**: 190–198

Kennedy AS, Raleigh JA, Perez GM, Calkins DP, Thrall DE, Novotny DB and Varia MA (1997) Proliferation and hypoxia in human squamous cell carcinoma of the cervix: first report of combined immunohistochemical assays. *Int J Radiat Oncol Biol Phys* **37**: 897–905

Leedy DA, Trune DR, Kronz JD and Weidner N (1994) Tumor angiogenesis, the p53 antigen, and cervical metastasis in squamous cell carcinoma of the tongue. *Otolaryngol Head Neck Surg* **111**: 417–422

Lyng H, Sundfør K and Rofstad EK (1997) Oxygen tension in human tumours measured with polarographic needle electrodes and its relationship to vascular density, necrosis and hypoxia. *Radiother Oncol* **44**: 163–169

Moriyama M, Kumagai S, Kawashiri S, Kojima K, Kakihara K and Yamamoto E (1997) Immunohistochemical study of tumor angiogenesis in oral squamous cell carcinoma. *Oral Oncology* **33**: 369–374

Murray JD, Carlson GW, McLaughlin K, Pennington M, Lynn M, DeRose PB, Williams JK and Cohen C (1997) Tumor angiogenesis as a prognostic factor in laryngeal cancer. *Am J Surg* **174**: 523–526

Nordsmark M, Overgaard M and Overgaard J (1996) Pretreatment oxygenation predicts radiation response in advanced squamous cell carcinoma of the head and neck. *Radiother Oncol* **41**: 31–39

Olive PL, Johnston PJ, Banáth JP and Durand RE (1998) The comet assay: A new method to examine heterogeneity associated with solid tumors. *Nature Medicine* **4**: 103–105

Raleigh JA, Calkins-Adams DP, Rinker LH, Ballenger CA, Weissler MC, Fowler Jr WC, Novotny DB and Varia MA (1998) Hypoxia and vascular endothelial growth factor expression in human squamous cell carcinomas using pimonidazole as a hypoxia marker. *Cancer Research* **58**: 3765–3768

Raleigh JA, Chou S-C, Arteel GE and Horsman MR (1999) Comparisons among pimonidazole binding, oxygen electrode measurements, and radiation response in C3H mouse tumors. *Radiat Res* **151**: 580–589

Rijken PFJW, Bernsen HJJA and Van der Kogel AJ (1995) Application of an image analysis system to the quantitation of tumor perfusion and vascularity in human glioma xenografts. *Microvasc Res* **50**: 141–153

Salven P, Heikillä P, Anttonen A, Kajanti M and Joensuu H (1997) Vascular endothelial growth factor in squamous cell head and neck carcinoma: expression and prognostic significance. *Modern Pathol* **10**: 1128–1133

Schlingemann RO, Dingjan GM, Emeis JJ, Blok J, Warnaar SO and Ruiter DJ (1985) Monoclonal antibody PAL-E specific for endothelium. *Labor Invest* **52**: 71–76

Schwartz G (1909) Über Desensibiliserung gegen Röntgen und Radiumstrahlen. *Munchener Medizinischen Wochenschrift* **24**: 1–2

Shpitzer T, Chaimoff M, Gal R, Stern Y, Feinmesser R and Segal K (1996) Tumor angiogenesis as a prognostic factor in early oral tongue cancer. *Arch Otolaryngol Head Neck Surg* **122**: 865–868

Thomlinson RH and Gray LH (1955) The histological structure of some human lung cancers and the possible implications for radiotherapy. *Br J Cancer* **9**: 539–549

Varia MA, Calkins-Adams DP, Rinker LH, Kennedy AS, Novotny DB, Fowler Jr WC and Raleigh JA (1998) Pimonidazole: A novel hypoxia marker for complementary study of tumor hypoxia and cell proliferation in cervical carcinoma. *Gynaecol Oncol* **71**: 270–277

Zätterström UK, Brun E, Willén R, Kjellén E and Wennerberg J (1995) Tumor angiogenesis and prognosis in squamous cell carcinoma of the head and neck. *Head and Neck* **17**: 312–318

*© 2000 Cancer Research Campaign* 
